# Supplementary material for: The Impact of Venous Invasion on the Postoperative Recurrence of pT1–3N0cM0 Gastric Cancer
Source: J Pers Med. 2023 Apr 26;13(5):734. doi: 10.3390/jpm13050734 (PMC10221240; doi:10.3390/jpm13050734)
Supplement: Supplementary file 1 [file jpm-13-00734-s001.zip › Figure S1.pdf]

Figure S1. Representative histopathology of venous invasion.

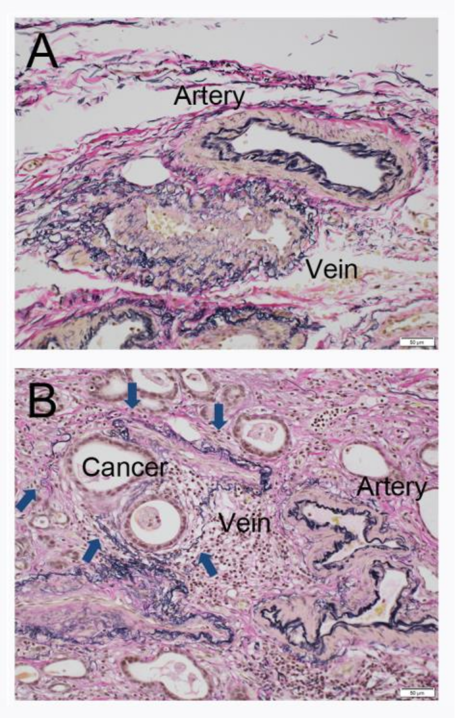

(A) Normal image of the artery and vein which run side by side. Elastic plates are stained black by Elastica van Gieson staining. (B) Venous invasion by cancer cells. Vein structure is destructed by cancer invasion, but the elastic plate (blue arrows) adjacent to the artery suggests adventitia of the vein.
