# Supplementary material for: The Impact of Venous Invasion on the Postoperative Recurrence of pT1–3N0cM0 Gastric Cancer
Source: J Pers Med. 2023 Apr 26;13(5):734. doi: 10.3390/jpm13050734 (PMC10221240; doi:10.3390/jpm13050734)
Supplement: Supplementary file 1 [file jpm-13-00734-s001.zip › Table S1.pdf]

Table S1. Adjuvant chemotherapy in relation to depth of tumor invasion and grade of venous invasion.

		Adjuvant chemotherapy			
		Yes	No	unknown	Total
VI grade*	v0	0	47	8	55
	v1	4	19	4	27
	v2	3	4	0	7
	v3	2	3	0	5
pT**	pT1	0	59	10	69
	pT2	1	7	1	9
	pT3	8	7	1	16

*Yes *vs.* No; $p < 0.001$ by Chi-square test with Yates' correction. **Yes *vs.* No; $p < 0.001$ by Chi-square test with Yates' correction. VI, venous invasion; pT, pathological depth of tumor invasion; v0, no venous invasion; v1, 1-3 invasions/slide; v2, 4-6 invasions/slide; v3, ≥ 7 invasions/slide. Filling-type of venous invasion in a macroscopically identifiable vein with a minor axis of ≥ 1 mm increased the VI grade by 1.
